# Use of human iPSC lines to model Alzheimer’s genetic risk and understand disease mechanism

**DOI:** 10.1002/alz.087340

**Published:** 2025-01-09

**Authors:** Alison M. Goate, Alexandra Münch, Carmen Romero Molina, Anna Podlesny‐Drabiniok, Grace Peppler, Francesca Garretti, Tulsi Patel, Hyo Lee, Edoardo Marcora

**Affiliations:** ^1^ Alzheimer Disease Research Center, New York, NY USA; ^2^ Icahn School of Medicine at Mount Sinai, New York, NY USA; ^3^ Ronald M. Loeb Center for Alzheimer’s disease, New York City, NY USA; ^4^ Icahn School of Medicine Mount Sinai, New york, NY USA; ^5^ Ronald M. Loeb Center for Alzheimer’s Disease, Friedman Brain Institute, Icahn School of Medicine at Mount Sinai, New York, NY USA; ^6^ Department of Genetics and Genomic Sciences, Icahn School of Medicine at Mount Sinai, New York, NY USA

## Abstract

**Background:**

Genome‐wide association studies (GWAS) have identified close to one hundred loci associated with Alzheimer’s disease (AD) risk. However, for most of these loci we do not understand the underlying mechanism leading to disease. Crispr genome editing in human induced pluripotent stem cells (hiPSCs) provides a model system to study the effects of these genetic variants in a disease relevant cell type.

**Method:**

We used integrative genomics to identify the cell types and genes that are most likely to explain the disease risk identified in GWAS and the direction of effect associated with elevated or reduced risk. We used crispr genome editing to modify expression of these candidate genes and tested the impact of modulating gene expression on myeloid cell function and AD associated phenotypes in vitro and in vivo.

**Result:**

AD risk alleles are enriched in myeloid cell enhancers but also in certain gene families. We have focused our functional studies on two such gene families: MS4A4A/MS4A6A and LACTB/B2. MS4A4A/MS4A6A are implicated in TREM2 signaling, and are major regulators of sTREM2 levels in cerebrospinal fluid (CSF) whereas LACTB/B2 are lactamases expressed in mitochondria. LACTB is the major modulator of succinyl carnitine levels, a metabolite in CSF associated with AD.

**Conclusion:**

iPSC provide powerful models for understanding the molecular mechanisms underlying genetic risk for AD

